# Genomic Insights of Antibiotic-Resistant *Escherichia coli* Isolated from Intensive Pig Farming in South Africa Using ‘Farm-to-Fork’ Approach

**DOI:** 10.3390/antibiotics14050446

**Published:** 2025-04-28

**Authors:** Shima E. Abdalla, Linda A. Bester, Akebe L. K. Abia, Mushal Allam, Arshad Ismail, Sabiha Y. Essack, Daniel G. Amoako

**Affiliations:** 1 Antimicrobial Research Unit, College of Health Sciences, University of KwaZulu-Natal, Durban 4000, South Africa; shimaeltayeb23@gmail.com (S.E.A.); abiaakebel@ukzn.ac.za (A.L.K.A.); essacks@ukzn.ac.za (S.Y.E.); 2 Biomedical Resource Unit, College of Health Sciences, University of KwaZulu-Natal, Durban 4000, South Africa; besterl@ukzn.ac.za; 3 Environmental Research Foundation, Westville 3630, South Africa; 4 Department of Genetics and Genomics, College of Medicine and Health Sciences, United Arab Emirates University, Al Ain P.O. Box 15551, United Arab Emirates; mushal.allam@uaeu.ac.ae; 5 Sequencing Core Facility, National Institute for Communicable Diseases, Division of the National Health Laboratory Service, Johannesburg 2193, South Africa; arshadi@nicd.ac.za; 6 Department of Biochemistry and Microbiology, Faculty of Science, Engineering and Agriculture, University of Venda, Thohoyandou 0950, South Africa; 7 Department of Pathobiology, University of Guelph, Guelph, ON N1G 2W1, Canada

**Keywords:** genomic epidemiology, *E. coli*, resistome, mobilome, antimicrobial resistance, pig production chain, South Africa

## Abstract

**Background/Objectives**: Intensive pig farming is a critical component of food security and economic activity in South Africa; however, it also presents a risk of amplifying antimicrobial resistance (AMR). This study provides genomic insights into antibiotic-resistant *Escherichia coli* (*E. coli*) circulating across the pork production chain, using a ‘farm-to-fork’ approach. **Methods**: A total of 417 samples were collected from various points along the production continuum, including the farm (n = 144), transport (n = 60), and abattoir (n = 213). *E. coli* isolates were identified using the Colilert-18 system, and their phenotypic resistance was tested against 20 antibiotics. Thirty-one isolates were selected for further characterization based on their resistance profiles and sampling sources, utilizing whole-genome sequencing and bioinformatic analysis. **Results**: The isolates exhibited varying resistance to critical antibiotics used in both human and animal health, including ampicillin (31/31, 100%), tetracycline (31/31, 100%), amoxicillin–clavulanate (29/31, 94%), chloramphenicol (25/31, 81%), and sulfamethoxazole–trimethoprim (10/31, 33%). Genetic analysis revealed the presence of resistance genes for β-lactams (*bla_EC_*, *bla_TEM_*), trimethoprim/sulfonamides (*dfrA1*, *dfrA5*, *dfrA12*, *sul2*, *sul3*), tetracyclines (*tetA*, *tetB*, *tetR*, *tet34*), aminoglycosides (*aadA*, *strA*, *aph* variants), and phenicols (*catB4*, *floR*, *cmlA1*), most of which were plasmid-borne. Virulome analysis identified 24 genes, including toxins and adhesion factors. Mobile genetic elements included 24 plasmid replicons, 43 prophages, 19 insertion sequence families, and 7 class 1 integrons. The *E. coli* isolates belonged to a diverse range of sequence types, demonstrating significant genetic variability. Further phylogenomic analysis revealed eight major clades, with isolate clustering by sequence type alongside South African environmental and clinical *E. coli* strains, regardless of their sampling source. **Conclusions**: The genetic complexity observed across the pork production continuum threatens food safety and may impact human health. These findings underscore the need for enhanced AMR monitoring in livestock systems and support the integration of AMR surveillance into food safety policy frameworks.

## 1. Introduction

Food-producing animals, including pigs, poultry, and cattle, are major reservoirs for foodborne pathogens, many of which are associated with significant human morbidity and mortality [1,2]. Globally, foodborne infections remain a persistent threat, accounting for millions of illnesses and numerous outbreaks annually [3,4].

*Escherichia coli* (*E. coli*) is a commensal bacterium typically present in the gastrointestinal tract of warm-blooded animals but capable of causing a range of intestinal and extraintestinal infections when pathogenic strains are involved [5,6]. Among foodborne pathogens, *E. coli* has gained prominence due to its increasing resistance to antibiotics of human and veterinary importance [7]. Antimicrobial resistance (AMR) in *E. coli* contributes significantly to the estimated 700,000 global annual deaths attributed to resistant infections, a figure projected to reach 10 million by 2050 in the absence of effective interventions [8,9]. Resistance in *E. coli* may arise through intrinsic mechanisms (e.g., efflux pumps, outer membrane impermeability) or acquired mechanisms such as chromosomal mutations and horizontal gene transfer via mobile genetic elements (MGEs), including plasmids, transposons, integrons, and bacteriophages [10,11]. These traits enable both commensal and pathogenic *E. coli* strains to act as reservoirs and vectors for antimicrobial resistance genes (ARGs) across the One Health interface [12].

In sub-Saharan Africa, particularly South Africa, pig farming remains a vital component of livestock agriculture and food security. However, the region faces growing concerns over antibiotic use and emerging resistance within intensive animal production systems [13,14]. Recent studies across Africa have reported high prevalence of MDR *E. coli* in livestock, underscoring the urgent need for localized surveillance strategies [15,16,17]. Despite increasing awareness, most AMR surveillance efforts in African livestock rely on traditional typing techniques, which lack the resolution of whole-genome sequencing (WGS) for discerning resistance mechanisms, phylogenetic relationships, and transmission pathways [18,19,20]. WGS provides unparalleled insights into the resistome, virulome, and mobilome of bacterial pathogens and is crucial for data-driven AMR risk assessment. To address these gaps, we employed a ‘farm-to-fork’ approach embedded within a One Health framework to characterize antibiotic-resistant *E. coli* circulating in the pig production continuum in the KwaZulu-Natal Province, South Africa. This study integrates phenotypic, genomic, and phylogenetic data to provide a holistic understanding of AMR risks associated with pork production systems and contributes critical regional data to support evidence-based policy and intervention design.

## 2. Results

### 2.1. Isolate Characteristics and Antibiotic Susceptibility Profiling

A total of 31 multidrug-resistant (MDR) *E. coli* isolates were selected for whole-genome sequencing and characterization based on their resistance profiles and representation across the pork production continuum comprising farm, transport, and abattoir stages. Of these, 19 isolates (61.3%) were derived from the farm environment, including 13 from pig fecal samples and 6 from slurry. Four isolates (12.9%) were recovered from the transport phase, evenly split between pre- and post-transport sampling. The remaining 8 isolates (25.8%) were obtained from abattoir-associated matrices, including cecal contents (n = 2), carcass rinsates (n = 2), carcass swabs (n = 2), and meat cuts (n = 2) (Table S1).

**Figure 1:**
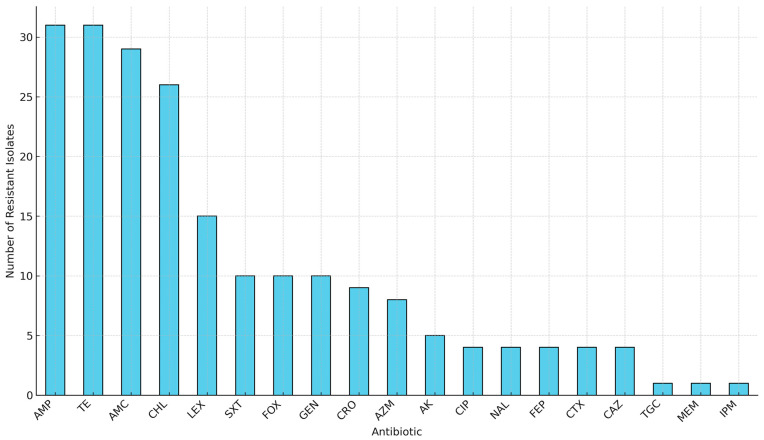
Overall antibiotic resistance frequencies among 31 MDR *E. coli* isolates from the farm-to-fork continuum. Key: AMP, Ampicillin; TE, Tetracycline; AMC, Amoxicillin-clavulanate; CHL, Chloramphenicol; LEX, Cephalexin; SXT, Sulfamethoxazole-trimethoprim; FOX, Cefoxitin; GEN, Gentamicin; CRO, Ceftriaxone; AZM, Azithromycin; AK, Amikacin; CIP, Ciprofloxacin; NAL, Nalidixic acid; FEP, Cefepime; CTX, Cefotaxime; CAZ, Ceftazidime; TGC, Tigecycline; MEM, Meropenem; IPM, Imipenem.

Phenotypic antibiotic susceptibility testing revealed that all 31 isolates (100%) exhibited resistance to ampicillin and tetracycline antibiotic classes commonly used in livestock production (Figure 1). High levels of resistance were also observed for amoxicillin–clavulanate (29/31, 93.5%) and chloramphenicol (25/31, 80.6%) (Figure 1). Moderate resistance to sulfamethoxazole–trimethoprim was observed in 10 isolates (32.3%). Low-level resistance was detected against critical last-resort agents such as tigecycline (1/31, 3.2%), imipenem (1/31, 3.2%), and meropenem (1/31, 3.2%) (Figure 1).

**Figure 2:**
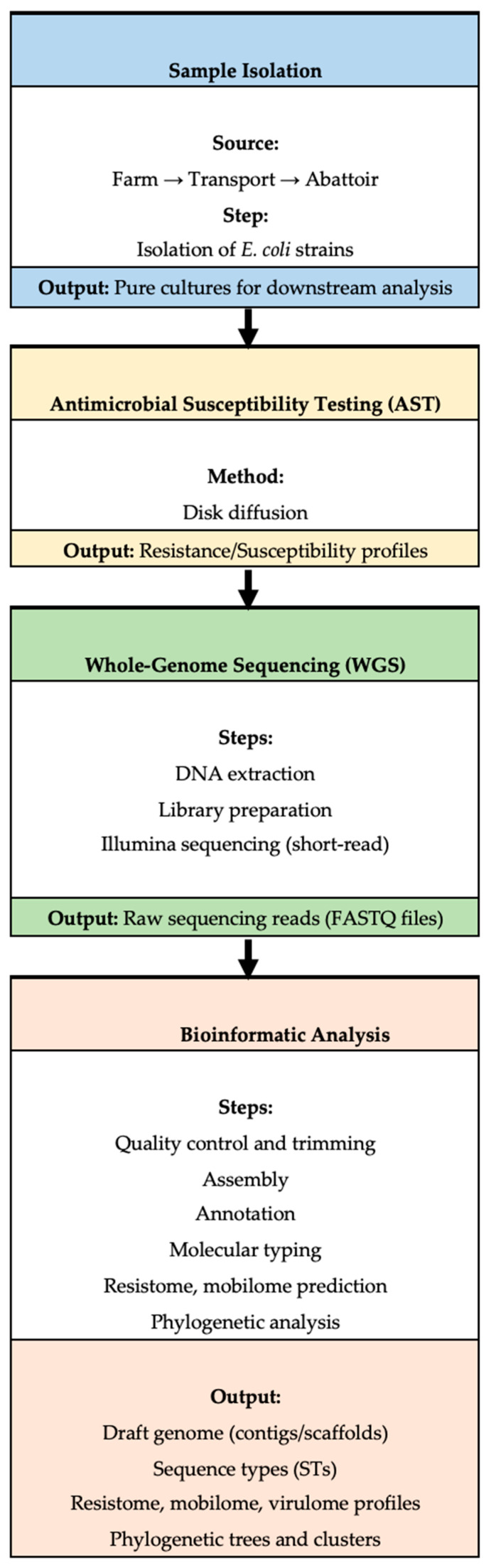
A workflow outlining the key steps in the study, including sample isolation, antimicrobial resistance (AMR) testing, whole-genome sequencing (WGS), and subsequent bioinformatic analysis.

Notably, several isolates displayed identical antibiogram profiles despite originating from distinct sources along the continuum, suggesting the possible circulation or persistence of clonally related resistant strains across production stages. While phenotypic resistance data for these isolates were previously reported in trend analyses [21], the current study extends this understanding through comprehensive genomic analyses of the resistome, virulome, mobilome, and phylogenetic relationships. Figure 2 provides a schematic overview of the study workflow, including sample isolation, antimicrobial susceptibility testing, WGS, and downstream bioinformatics pipelines.

### 2.2. Genomic Characteristics

The genomic features of the 31 *E. coli* isolates are summarized in Supplementary Table S2. The total assembled genome sizes ranged from 4.7 to 6.1 megabases (Mb), consistent with typical *E. coli* genome architecture. The GC content varied narrowly between 50.2% and 50.8%, reflecting a conserved base composition across isolates.

Assembly quality metrics further supported the robustness of the sequencing data, with most assemblies meeting the established thresholds for downstream genomic analysis. These metrics reflect the expected diversity in assembly outcomes based on sequencing depth, strain-specific genome complexity, and repeat content. All genomes passed quality control filters and were deemed suitable for high-resolution comparative analyses of AMR, virulence, and phylogenomic structure.

### 2.3. Antibiotic Resistance Gene Analysis

**Figure 3:**
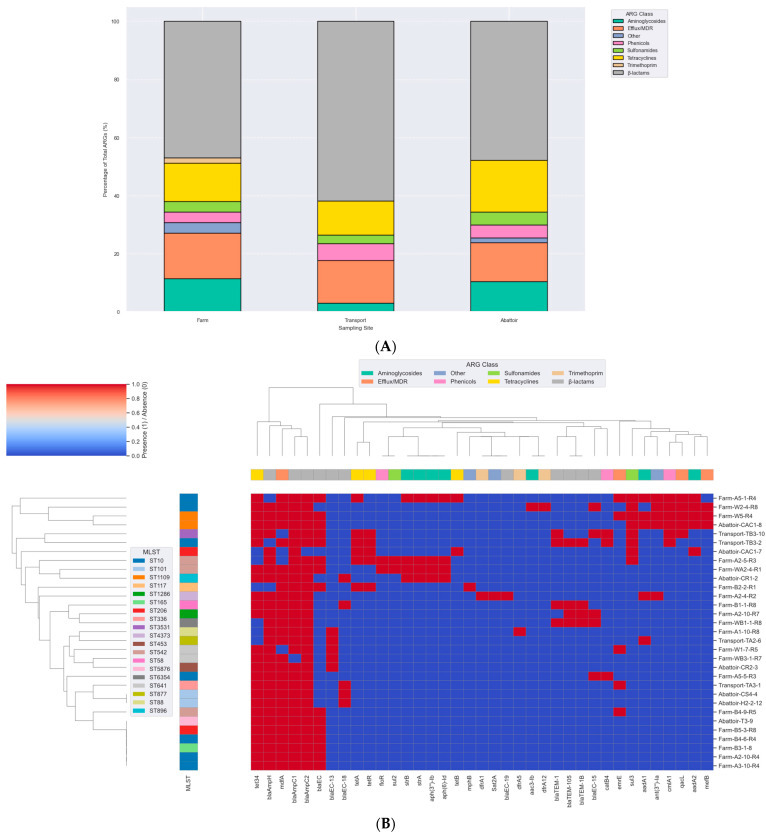
(**A**) Percentage distribution of ARG classes across 31 *E. coli* isolates. The stacked bar chart depicts the relative proportions of ARG classes detected in isolates obtained from farm (n = 19), transport (n = 4), and abattoir (n = 8) sources. Resistance genes were categorized into functional classes, including β-lactams, tetracyclines, aminoglycosides, sulfonamides, trimethoprim, streptothricin, and efflux/multidrug resistance (MDR) determinants. Each bar represents the percentage contribution of each ARG class to the total ARGs identified at that site, highlighting variation in resistome composition across different stages of the pork production system. (**B**) Clustermap of antimicrobial resistance gene (ARG) profiles across MDR *Escherichia coli* isolates from the pork production continuum in South Africa. Hierarchical clustering was performed based on presence/absence of ARGs, with isolates grouped according to similarity in resistome composition. Each column represents a specific ARG, while each row corresponds to an isolate labeled by its sampling source (Farm, Transport, Abattoir). The color intensity indicates gene presence (red) or absence (blue). The top color band denotes the functional class of each ARG, including β-lactams, tetracyclines, sulfonamides, aminoglycosides, trimethoprim, streptothricin, and efflux/MDR resistance determinants.

WGS revealed a rich and diverse resistome across all *E. coli* isolates, with resistance determinants spanning major antibiotic classes across sampling sites. β-lactam resistance was the most prominent feature, characterized by the widespread presence of chromosomal and plasmid-encoded β-lactamase genes (Figure 3A and Table S1). In addition, ESBL genes *bla_TEM-1_*, *bla_TEM-1B_*, and *bla_TEM-105_* were detected. Aminoglycoside resistance was conferred by an array of modifying enzyme genes, indicating frequent acquisition of MGEs associated with this class. Resistance to tetracyclines was predominantly mediated by *tet34*, *tetA*, and *tetB*, while resistance to sulfonamides and trimethoprim was driven by *sul2*, *sul3*, *dfrA1*, *dfrA5*, and *dfrA12*. Additional resistance determinants included *catB4*, *floR*, and *cmlA1* for phenicols, and macrolide resistance genes (Figure 3B).

Of particular note, several efflux system genes were recurrently identified and are known to mediate low-level MDR across diverse antibiotic classes including macrolides, fluoroquinolones, chloramphenicol, tetracyclines, and certain aminoglycosides (Figure 3A,B). The distribution of ARGs was largely conserved across the production continuum. Farm isolates harbored a core resistome comprising *aadA*, *strA*, and *strB* in some samples, with the additional detection of *sul3*. Transport isolates exhibited comparable resistance profiles, though with slightly fewer ARGs overall. Abattoir isolates retained key resistance determinants seen upstream (Figure 3B). The phenotypes were not corroborated by ARGs in some cases, as some isolates exhibited resistance to antibiotics in the absence of known associated resistance determinants (Table S1). For example, the mechanism of β-lactam resistance in some isolates, such as WB1-1-R8, was not detected in the ResFinder and CARD databases (Table S1).

The quinolone resistance-determining regions (QRDRs) were investigated for chromosomal amino acid substitutions. Substitutions were observed in the chromosome-borne DNA gyrase (*gyrA*-S83L, D87N, E678D, S828A and *gyrB*-D185E) and topoisomerase IV (*parC*-S80I, E475D, A620V and *parE*-I136V), mediating fluoroquinolone resistance (Table 1). Similarly, amino acid substitutions in *acrA* (T104A), *acrR* (A212S, N214T), and *marB* (L27P), along with the presence of *tetA* and *tolC* genes, were identified as contributing to tigecycline resistance in the isolate resistant to this antibiotic (Table 1).

### 2.4. Mobilome (Plasmids, Insertion Sequences, Prophages, and Integrons) Analysis

PlasmidFinder revealed 24 different plasmid replicon types. Farm isolates carried a diverse range of plasmid replicons including Col(MG828), IncFIB(AP001918), IncFII, IncX1, and IncY—all associated with the transmission of resistance genes. In transport isolates, plasmid replicons such as ColRNAI, IncFIB(AP001918), IncFII, and IncX1 were also present. The similarity of these plasmid replicons between farm and transport isolates suggests a potential continuity of resistance mechanisms during transport. Abattoir isolates carried plasmid replicons similar to those found in the farm and transport stages, including ColRNAI, IncFIB(AP001918), IncFII, and IncX1. This consistent presence of plasmid replicons across the continuum supports the hypothesis of plasmid-mediated gene transfer throughout the pig production chain (Table S1).

**Figure 4:**
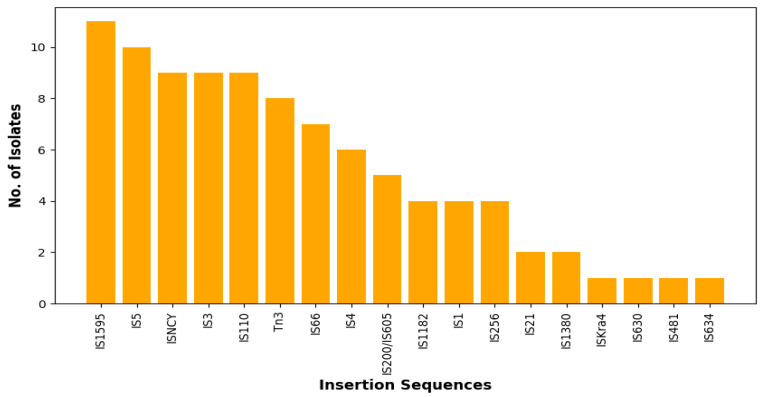
Total number of each predicted insertion sequence (IS) families via the ISFINDER database (https://isfinder.biotoul.fr/ accessed on 15 June 2024).

Nineteen insertion sequence (ISs) families were found in all the genomes with the most predominant being IS1595 (35.5%), followed by IS5 (32.2%) (Figure 4). In general, there was great diversity of the IS families irrespective source or sequence type, albeit there were few instances where this was not the case. For example, two *E. coli* isolates from the farm belonging to ST10 (A2-10-R4, A3-10-R4) had the same set of ISs (ISNCY, IS4 and IS1595) whilst two isolates from the abattoir belonging to ST1109 (CAC1-8, W-5-R4) also encoded the same ISs (Tn3, IS66 and IS5). The PHAge Search Tool revealed 43 intact phages, the most predominant of which was Entero_lambda found in 25 isolates, followed by Entero_P88, Salmon_Fels_2, and Shigel_SfII found in 15, 15, and 13 of the isolates, respectively (Supplementary Table S3). None of the prophages carried ARGs.

Class 1 and 2 integrons were found in the isolates, with Class 1 being the dominant (Table 2). Class 1 integrons were identified in 7 (22.6%) of the isolates found on the farm (n = 3) and abattoir (n = 4). In456 was the most frequently identified class 1 integron with resistance gene cassettes encoding resistance to aminoglycosides (n = 2) and chloramphenicol (n = 1) and bracketed by the 1S256 insertion sequences and TnAs1 transposon (Table 3). The most frequently identified gene cassette combination was *aadA1:aadA2: cmlA1*, found in two integrons, including In456 and In649 (Table 2). Similar integron types with identical gene cassettes were identified in isolates from different clonal types and sampling sites. For example, isolates from the farm A5-1-1R4 (ST10), W-5-R4 (ST1109), and abattoir CAC1-8 (ST1109) had the In456 integron type with an identical gene cassette (*aadA1:aadA2:cmlA1*). Cassette arrays did not follow clonal lineages or sources (farm and abattoir), while isolates belonging to the same STs had different gene cassettes (Table 2). The unique class 2 integron with the resistance gene cassettes (*dfrA1::aadA1::sat2*) was found in isolate A2-4-R2(ST4373) from the farm. Some ARGs were mostly co-carried on MGEs (integrons or associated with insertion sequences and/or transposons) (Table 3). The *bla*_TEM-1B_ gene was commonly associated with *Tn3* transposon. The *Tn3* was also associated with tetracycline resistance genes in some instances. The insertion sequence (IS256) was associated with the *sul3* gene (Table 3). The resistance genes and MGEs in the *E. coli* isolates were closely related (98–100% similarity) to target sequences in the GenBank database, with most hits being plasmids with hosts from the *Enterobacterales* family (Table 3).

### 2.5. Virulome in the E. coli Isolates

**Figure 5:**
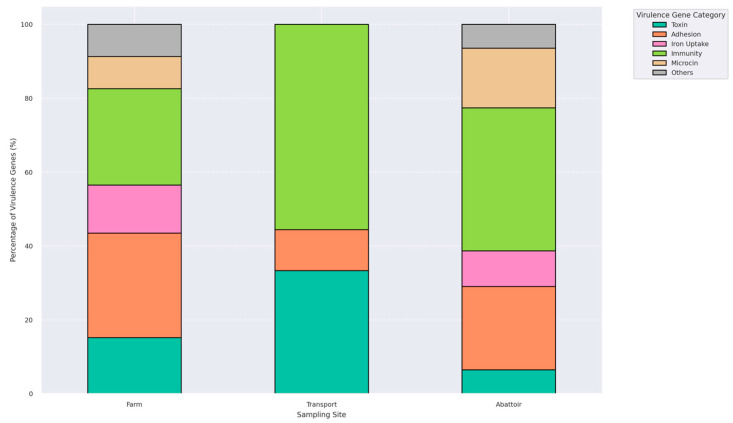
A plot illustrating the relative proportions of six functional virulence gene categories: toxins, adhesion factors, iron uptake systems, immune evasion mechanisms, microcins, and others, identified in isolates from farm, transport, and abattoir environments. Each bar represents the total virulence gene composition per interface, normalized to 100%, highlighting shifts in virulome across the farm continuum.

Virulome profiling identified 24 distinct virulence-associated genes among the 31 *E. coli* isolates, with 77.4% (24/31) carrying at least one virulence determinant (Table S4). A total of 20 isolates (64.5%) harbored multiple virulence genes. These genes were grouped into functional categories: toxins (*astA*, *vat*, *pic*, *capU*, *cba*, *cma*), adhesion factors (*lpfA*, *tsh*, *eilA*, *iha*, *stx2A*, *stx2B*, *stx2*), iron acquisition systems (*iroN*, *ireA*), immune evasion elements (*iss*, *gad*, *air*, *katP*), microcins (*mchB*, *mchC*, *mchF*, *mcmA*), and one secretion-associated gene (*aaiC*) (Figure 5). The most commonly detected virulence genes were *iss* (n = 16), *lpfA* (n = 13), *astA* (n = 12), and *gad* (n = 9). MGEs associated with these virulence genes are presented in Table 4. Farm isolates exhibited a broad virulence gene repertoire, including *gad*, *astA*, *iroN*, *iss*, and *mchF*. Transport isolates exhibited the lowest diversity in virulence gene categories compared to those from the farm and abattoir stages (Figure 5).

### 2.6. Multilocus Sequence Typing (MLST) and Phylogenomic Insights

**Figure 6:**
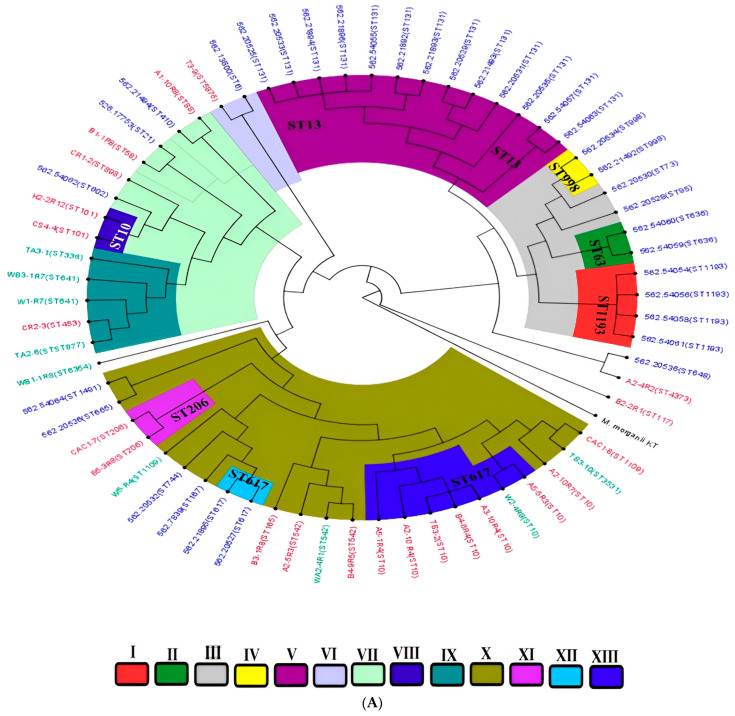

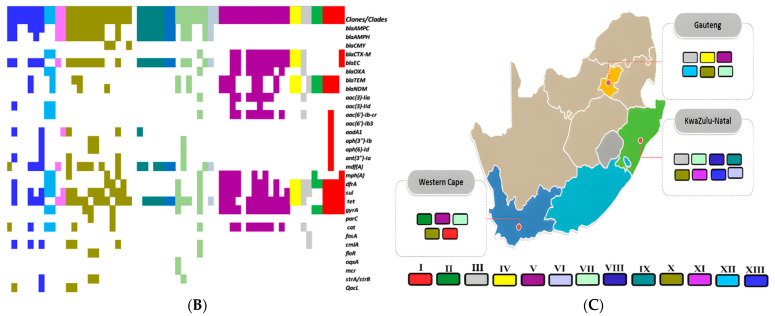
(**A**) A phylogenetic tree depicting the relationships among *E. coli* isolates from this study alongside South African isolates from diverse One Health sources. The isolates are clustered by sequence types (STs), with tip color annotations indicating their origins: animal-source isolates (purple), human clinical isolates (blue), and environmental isolates (green). Clades are designated **I–XIII**, with distinct branch colors corresponding to each clade for ease of visualization. (**B**) A heatmap illustrating the distribution of antimicrobial resistance genes (resistome) across different clades (**I–XIII**). The color intensity represents the presence or absence of specific resistance genes within each isolate. (**C**) The geographic distribution of clades (**I–XIII**) across South African provinces, with colors matching those in the phylogenetic tree. Clade colors are consistent with those shown in the phylogenetic tree (**A**), facilitating comparison between genetic clustering and spatial distribution patterns.

In silico multilocus sequence typing identified substantial genetic diversity among the *E. coli* isolates, with representation across some distinct sequence types (STs) (Figure 3B). Farm-derived isolates included ST10, ST58, ST117, ST542, and ST4373, among others, with ST10 appearing most frequently (Table S1). Transport-stage isolates were characterized by unique sequence types such as ST877, ST336, ST3531, and ST10. Abattoir isolates showed continued diversity, comprising ST206, ST101, ST898, ST1109, and ST5876 (Figure 3B; Table S1). Whole-genome SNP-based phylogenetic analysis delineated eight major clades (Clades I–VIII), comprising isolates from this study and publicly available *E. coli* genomes from South Africa (Figure 6A). Isolates were clustered by sequence type rather than source, with study isolates co-localizing with human clinical and environmental strains. For instance, ST206 isolates formed a distinct clade (Clade XI), while ST10, ST1109, and ST101 were found in clusters containing both animal- and human-derived strains. Isolates from different provinces (KwaZulu-Natal, Gauteng, and Western Cape) were interspersed across clades, indicating no clear geographic partitioning. No clade-specific associations were observed between resistomic profiles and phylogenetic structure (Figure 6B). Resistance determinants appeared distributed across unrelated lineages, consistent with horizontal gene transfer mechanisms. The phylogenetic tree, resistome heatmap, and geographic distribution of clades are shown in Figure 6A–C.

## 3. Discussion

This study describes the genomic epidemiology of antibiotic-resistant *E. coli* isolated from an intensive pig production continuum in uMgungundlovu District, KwaZulu-Natal, South Africa, using the farm-to-fork approach (farm–transport–abattoir). Overall, the isolates showed high resistance against critically important antibiotics for humans and animals, threatening available antibiotic arsenals for treatment. The myriad of antibiotic resistance genes and mobile genetic support encoded confirms that pigs and pork could serve as potential reservoirs for antibiotic resistance. The high occurrence of virulence genes detected in the continuum also challenges food safety and is a potential risk to human health. The diversity of clones indicates the complexity of antibiotic-resistant *E. coli* within the continuum, highlighting the need for stricter sanitary conditions to prevent their transmission along the production chain. The use of antibiotics in pork production, particularly in animal feed for growth promotion and prophylaxis, has long been recognized as a major driver of antimicrobial resistance (AMR) in livestock systems [3,22]. Continuous exposure to sub-therapeutic antibiotic concentrations creates selective pressure that fosters the emergence and dissemination of resistant bacterial populations. This not only amplifies the local resistome but also facilitates horizontal gene transfer among commensal and pathogenic bacteria [23]. This study highlights the critical need for responsible antibiotic stewardship in animal husbandry and support the implementation of stricter regulations governing antibiotic use in livestock in South Africa. Such measures are essential to curb the selection of multidrug-resistant *E. coli* strains that can spread across the farm-to-fork continuum and threaten public health.

### 3.1. The Genetic Basis of Antibiotic Resistance and Their Mobile Genetic Support

Resistance in this study was related to several acquired antibiotic resistance genes, chromosomal mutations, and efflux genes. β-lactamases were detected in all the isolates from different sites and sources in the pork production continuum, highlighting the wide distribution of these resistant genes in healthy pigs and their products, which is associated with the high use of antimicrobials [24]. As it is normally expressed constitutively in *E. coli* [25], the plasmid-mediated *AmpC* (*bla*_AmpC1_ and *bla*_AmpC2_) was found in all the isolates (100%) in the current study. The hyperproduction of constitutively produced *AmpC* and plasmid-mediated *Amp*C β-lactamases that hydrolyze β-lactams antibiotics, including third- and fourth-generation cephalosporins, have been detected in pigs worldwide [26]. *AmpC*-producing bacteria were observed in clinical isolates initially, but then these bacteria were observed in companion animals as well as livestock, supporting the hypothesis that food-producing animals are the source and reservoir of these AmpC-producing bacteria [27]. The class C β-lactamase *bla*_EC_ [11] were found in all the isolates. *bla_EC_*_-15_ and *bla*_EC-18_ have been reported previously in *E. coli* isolated from cattle feces in Canada [28].

ESBLs hydrolyze monobactams and broad-spectrum cephalosporins [29]. ESBL-positive *E. coli* isolated from pigs have been reported in China, Korea, and Portugal [30,31,32], with recent studies continuing to highlight their public health relevance. In the current study, *bla*_TEM_ (*bla*_TEM-1B_ and *bla*_TEM-105_) was found in four isolates, three of which were farm-derived and exhibited resistance to all tested cephalosporins. These findings align with earlier reports of *bla*_TEM_-harboring *E. coli* from pig farms in China, Korea, France, and more recently, from intensive farming systems in Africa, underscoring the ongoing threat posed by ESBL-producing strains in the pig production sector [31,33,34,35]. TEM-type derivatives, *bla*_TEM-1B_ and *bla*_TEM-2_, considered the most common β-lactamase found in *Enterobacterales of* both human and animal origin [31], are known to be contained within a *TniA* group of transposons or fragments from one of them [36]. In this study, all *bla*_TEM_ genes were associated with *Tn3* transposase carried on plasmids (Table 3). The phenomenon of isolates carrying the same ESBL genes but showing different phenotypes, as seen in this study, has been previously documented [10]. Most ESBL-producing bacteria are multi-resistant, exhibiting additional resistance to non-cephalosporin antibiotics such as fluoroquinolones and aminoglycosides [37,38]. Therefore, the frequent use of broad-spectrum cephalosporins for prophylaxis or treatment can facilitate the emergence of MDR *E. coli*. In 2011, the Panel on Biological Hazards (BIOHAZ) highlighted the rapid emergence of ESBL/*Amp*C-producing *Enterobacterales* in food-producing animals as a major public health concern [39].

With the long-standing and extensive use of tetracycline in humans and animals, the tetracycline resistance genes, which can be horizontally transferred, have been intensively studied [10]. The *tet34* gene, which codes for an enzyme that inactivates tetracycline, predominated in this study, although to our knowledge, *tet34* has not been reported in pigs. *tetA* and *tetB* coding for energy-dependent efflux proteins, which help bacteria pump tetracycline out of the cell [10,40], were also detected. The *tetA* and *tetB* genes were adjacent to transcriptional repressor gene *tetR*(*A*) and *tetR*(*B*), respectively. Except for one farm isolate (A2-5R3), which encoded *tetA* found on the chromosome and mediated by insertion sequence IS1, all the other *tetA* and *tetB* were conjugated with transposons and found in plasmids which may mediate their transfer [41]. More so, the presence of the *mdfA* gene belonging to the major facilitator superfamily (MFS), whose overexpression has been reported in multidrug-resistant *E. coli* strains isolated from patients [42], seems to contribute to the tetracycline resistance.

Since chloramphenicol is not widely used in veterinary medicine, some studies related their high resistance rate to the multi-resistant efflux system and/or co-selection with structurally unrelated compounds [43,44,45]. In this study, resistance to chloramphenicol was mainly due to gene *cmlA1* conjugated to different MGEs, such as insertion sequences (IS1, IS26, IS256), *Tn3* and class 1 integrons (In 456, In649, In774, In127), and co-carried with ARGs to aminoglycosides, macrolides, sulfonamides, trimethoprim, and tetracycline. The *cmlA1* in this study was carried by plasmids, suggesting its possibility for horizontal transfer. The *catB* gene that encodes acetyltransferases that inactivate chloramphenicol was only detected in isolates from transport vehicles. The efflux genes *floR*, known to mediate resistance to chloramphenicol, were found on the farm only. The *floR* was associated with IS91 found in the chromosome with the closest nucleotide homology with *E. coli* strain AH25 chromosome, complete genome (CP055256.1). The presence of these three genes (*cmlA1*, *catB*, and *floR)* in pigs was previously reported in Australia and China [46,47]. The presence of these resistance genes in the plasmid facilitates their transmission. The co-selection of chloramphenicol resistance genes with other resistance genes belonging to other antibiotic categories may explain its high resistance rate even without selection pressure.

Previous pig studies have reported sulfamethoxazole *sul* and trimethoprim *dfrA* resistance genes [48,49]. Resistance to sulfonamides in *E. coli* frequently results from acquiring an alternative dihydropteroate synthase (DHPS) gene *sul*. Three alternative sulfonamide resistance DHPS genes [*sul1*, *sul2*, and *sul3*] in Gram-negative bacteria have been documented [50], and two genes (*sul2* and *sul3*) were reported in this study. Both *sul* and *dfrA* genes were found in co-occurrence with resistant genes belonging to other antibiotic classes. They were also found in association with different MGEs, suggesting their easy mobilization.

Genes responsible for aminoglycoside resistance were also found in this study. These genes are transmissible, encoded on conjugative plasmids, and often linked to resistance to other antimicrobials [51]. The resistance determinants (*aadA*, *strA*, *strB*, *aph(3*″*)-Ib*, *aph(6)-Id*) found in this study were also previously detected in *E. coli* isolated from pigs in the United States [52]. The *aadA1* and *aadA2* genes found in this study were often located in integrons (class1 and class2) carrying a gene cassette involving genes responsible for chloramphenicol and trimethoprim resistance (Table 2). The presence of integrons containing resistance gene cassettes contributes to the presence of MDR isolates [53].

Macrolides generally show modest potency against *Enterobacterales* [54]. Different macrolide resistance genes have been reported, causing resistance through various pathways. This can be through macrolide inactivation, phosphorylation (*mph A*, *mph B*), and transferable genes (*msr A*, *mef A or mef B)* encoding macrolide-efflux pumps [55]. The presence of the ethidium multidrug resistance protein E (*emr*E) gene which is a member of the small multidrug resistance (SMR) efflux protein family [56] in isolates phenotypically resistant to azithromycin may suggest the contribution of the gene to azithromycin resistance [57].

*E. coli* resistance to fluoroquinolones is primarily mediated by a specific mutation in the quinolone resistance-determining region (QRDR) within subunits constituting topoisomerase II (*gyrA* and *gyrB*) and IV (*parC* and *parE*), which are involved in DNA replication. These mutations are known to reduce the binding efficiency of fluoroquinolone drugs, thereby leading to resistance to these drugs [10]. This study found mutations in the six isolates showing phenotypic resistance to fluoroquinolones (Table 1). Mutations in *gyrA* and *parC* from *E. coli* isolated from pigs have been previously reported [57,58,59]. These mutations’ presence enables bacteria to acquire more plasmids, which may contribute to acquiring other resistance genes. Only one farm isolate (B2-2-R1) showed phenotypic tigecycline resistance. The presence of chromosomal mutations in the genes (*acrA*, *acrR*, and *marB*) and the presence of *tet*A and *tol*C genes found in this isolate, which mediated resistance to the tigecycline, has been reported previously [60,61]. The persistence of plasmid replicons such as Col(MG828), IncFIB(AP001918), IncFII, and IncX1 across different stages of the pig production chain points to the potential role of plasmids in the dissemination of resistance genes across the continuum.

This study demonstrated a generally strong correlation between phenotypic resistance profiles and the presence of corresponding antimicrobial resistance genes (ARGs) detected through whole-genome sequencing; however, notable phenotypic–genotypic discrepancies were also observed in some isolates. These inconsistencies may be attributed to several factors, including the presence of novel or uncharacterized resistance mechanisms not captured by current databases, gene silencing or low expression levels that do not manifest phenotypically, and potential sequencing or assembly limitations that could lead to the partial or incomplete detection of ARGs [10,62]. Additionally, the phenotypic expression of resistance may be influenced by regulatory mutations, efflux pumps, or synergistic effects among multiple resistance determinants. These findings underscore the complexity of AMR expression and highlight the need for continuous updates to susceptibility testing guidelines, resistance gene databases, and improvements in bioinformatic tools to enhance genotype–phenotype correlation in AMR surveillance.

### 3.2. Virulome Analysis

The virulome landscape of *E. coli* isolates recovered from the pork production system revealed a concerning convergence of diversity, functional complexity, and ecological adaptation. Over three-quarters of the isolates (77.4%) encoded at least one virulence determinant, and nearly two-thirds (64.5%) harbored multiple functional genes, underscoring the latent pathogenic potential of asymptomatically carried strains within livestock systems. This polygenic virulence architecture mirrors recent findings from Australia, Mexico, and sub-Saharan Africa, where livestock-associated *E. coli* exhibits similar profiles [62,63], highlighting the global relevance of these silent zoonotic reservoirs. Functionally, virulence genes were stratified across six primary categories: adhesion (*lpfA*, *eilA*, *tsh*, *stx2A/B*), toxin production (*astA*, *vat*, *capU*), immune evasion (*iss*, *gad*, *katP*), iron acquisition (*iroN*, *ireA*), microcins (*mchB*, *mchC*, *mchF*, *mcmA*), and secretion systems (*aaiC*). The dominance of *iss*, *astA*, and *lpfA* across interfaces points to enhanced mucosal colonization and immune evasion potential hallmarks of extraintestinal pathogenic *E. coli* (ExPEC) [5]. Strikingly, a single isolate from the abattoir carried *stx2*, a defining toxin of Shiga toxin-producing *E. coli* (STEC), marking a rare but consequential event with implications for hybrid pathotype emergence at the human–animal interface.

What elevates the virulome risk landscape is not only the diversity of virulence traits but also their mobilization potential. Many genes, particularly *gad*, *tsh*, and *eilA*, were flanked by mobile genetic elements (MGEs), including insertion sequences (ISAs1-like, IS3 family), transposases, and class 1 integrons. The physical proximity of virulence loci to MGEs with some chromosomally integrated, others plasmid-borne suggests that horizontal transfer under selective pressure remains a viable evolutionary pathway [52]. This genetic plasticity aligns with the growing body of evidence that livestock ecosystems serve not only as reservoirs of AMR but also as incubators for virulence convergence. Mechanistically, the association of ExPEC-related genes with MGEs capable of mobilization raises the specter of genomic co-selection, wherein antimicrobial use may inadvertently drive the spread of virulence factors. This dual burden of virulence and resistance has been increasingly reported in clonal lineages circulating in both clinical and foodborne settings [63], reinforcing the interconnected nature of One Health risks. While the detection of *stx2* was limited to a single isolate, its presence, even in the absence of disease, reaffirms the limitations of symptom-based risk assessment in food systems. From a One Health perspective, these results underscore the silent carriage of potentially pathogenic *E. coli* within the pork production continuum. The fact that many of these virulence signatures were identified in pre-slaughter stages emphasizes the need for upstream surveillance. Current food safety paradigms anchored in overt clinical signs may underestimate the risk posed by asymptomatic carriers of highly functional virulence machinery.

Taken together, our findings highlight the value of virulome profiling as an essential complement to AMR surveillance. The integration of functional genomics into risk assessment frameworks offers a more nuanced understanding of pathogen emergence and dissemination. As livestock systems intensify, genomic surveillance must evolve from reactive to anticipatory, enabling the early detection of high-risk strains before they reach the food chain. Future policy should prioritize molecular monitoring of virulence traits alongside resistance determinants to inform risk-based inspections, guide mitigation efforts, and prevent the silent transmission of hybrid and high-consequence *E. coli* pathotypes.

### 3.3. Multilocus Sequence Typing and Phylogenomic Analysis

The sequence type (ST) diversity among *E. coli* isolates across the pork production continuum revealed both ecological segregation and genetic connectivity. While distinct STs were observed within each production interface (farm, transport, and abattoir, respectively), the recurrence of lineages such as ST10, ST206, and ST101 across multiple stages suggests persistence and potential intra-system dissemination. This overlap is indicative of longitudinal transmission pathways, possibly facilitated by shared handling practices, environmental persistence, or bacterial fitness traits that enable survival across varied niches. The predominance of ST10, a member of the clonal complex CC10, is particularly noteworthy. This lineage has been frequently reported in pig-associated *E. coli* isolates from Denmark, Australia, Ireland, and Portugal [32,59], and is recognized as the dominant sequence type in swine populations across several European contexts, including Germany, Denmark, Ireland, and Spain [64]. ST10 and its related clonal complex members exhibit multidrug-resistant (MDR) phenotypes and are associated with both intestinal and extraintestinal infections in animals and humans [65]. Its detection in this study reinforces CC10′s adaptive success and zoonotic relevance and further positions it as a critical lineage at the livestock–human interface.

Beyond clonal assignment, phylogenomic reconstruction of isolates from South Africa revealed eight supported clades encompassing isolates from humans, animals, and environmental samples. Importantly, several clades showed phylogenetic continuity across these source categories, suggesting the inter-compartmental circulation of certain *E. coli* lineages. This finding is consistent with recent global reports that document the erosion of ecological boundaries in *E. coli* populations, where the same clone circulates across hosts and environments via food, water, or contact transmission. Notably, several clinical isolates (n = 10) from Gauteng and the Western Cape clustered closely with isolates from this study, indicating likely spillover from different sources. Such convergence strongly implicates the food value chain in the broader *E. coli* transmission network and highlights the limitations of treating foodborne *E. coli* as taxonomically distinct from clinical counterparts.

These data provide genomic evidence of One Health connectivity, where livestock-associated MDR *E. coli* lineages, especially those within CC10, circulate between animals, humans, and the environment. This dynamic not only challenges siloed surveillance approaches but also reinforces the urgency for integrated genomic surveillance frameworks that operate across sectors. Importantly, the phylogenetic proximity of foodborne and clinical strains highlights the potential for livestock-derived *E. coli* to acquire or co-circulate with human-adapted virulence and resistance elements, accelerating the emergence of high-risk clones. Overall, MLST and high-resolution phylogenomics expose the hidden population structure of *E. coli* circulating in the pork production ecosystem. They also reveal a mosaic of shared lineages that blur the boundaries between food safety and clinical microbiology.

This study was limited by the small number of sequenced isolates, primarily due to resource constraints. Although the dataset provided meaningful insights into the genomic diversity of *E. coli* across the farm-to-fork continuum, the limited sample size may not fully reflect the broader population structure or capture the full extent of strain transmission dynamics within and between production stages. This underscores the need for increased investment in genomic surveillance capacity in low- and middle-income settings, where high-resolution data are critical for effective antimicrobial resistance (AMR) monitoring [19,20]. Additionally, it highlights the importance of designing future studies with larger and more representative sampling frameworks spanning diverse farms, production systems, and geographic regions to generate data that can better inform national AMR control strategies.

More so, to strengthen surveillance and policy planning, future research should incorporate the whole-genome sequencing of a broader set of isolates alongside detailed epidemiological metadata. This approach would enhance the ability to detect emerging high-risk clones, identify resistance hotspots, and trace the spread of resistant bacteria across the animal–human–environment interface. Such evidence is essential for shaping data-driven policies on antimicrobial stewardship in livestock production, improving biosecurity protocols at slaughterhouses, and informing cross-sectoral One Health interventions.

## 4. Materials and Methods

### 4.1. Ethical Statement

Ethical approval was received from the Animal Research Ethics Committee (Reference: AREC/007/018) and the Biomedical Research Ethics Committee (Reference: BCA444/16) of the University of KwaZulu-Natal. The study was further approved by the South African National Department of Agriculture, Forestry, and Fisheries (Reference: 12/11/1/5).

### 4.2. Study Site and Sample Collection

The study was a longitudinal study conducted in the uMgungundlovu District, KwaZulu Natal, South Africa, over eighteen weeks from September 2018 to January 2019. The main sampling site was an intensive pig farm. Samples were collected from different points along the pork production system of this farm, as stipulated by the World Health Organization Advisory Group on Integrated Surveillance of Antimicrobial Resistance (WHO-AGISAR) guidelines [66]. A total of 417 samples were collected across the farm-to-fork continuum, including farm (n = 144), transport (n = 60), and abattoir (n = 213) samples, as previously described [21].

### 4.3. E. coli Isolation, Confirmation, and Antibiotic Susceptibility Testing

A total of 1044 *E. coli* isolates were putatively identified during enumeration using the Colilert ^TM^ 18 Quanti-Tray/2000 system (IDEXX Laboratories (Pty) Ltd., Johannesburg, South Africa), followed by phenotypic confirmation on eosin methylene blue (EMB) [67]. For *E. coli* confirmation, DNA was extracted from these isolates using the boiling method and confirmed by real-time polymerase chain reaction (PCR), targeting the *uidA* gene as previously described [21]. All reactions were performed on a Quant Studio 5 (Thermo Fischer Scientific, MA, USA). *E. coli* ATCC**^®^** 25,922 was used as a positive control, while the reaction mixture with no DNA (replaced with nuclease-free water) was used as a negative template control. Isolates confirmed as *E. coli* were tested for susceptibility to 20 antibiotics using the disk diffusion method following WHO-AGISAR recommendations and CLSI 2020 guidelines, as previously published [21].

### 4.4. Selection of Isolates, Whole-Genome Sequencing, and Bioinformatic Analysis

#### 4.4.1. Isolate Selection

A total of thirty-one non-duplicate *Escherichia coli* isolates were selected from a larger pool of phenotypically multidrug-resistant (MDR) strains recovered during routine antimicrobial surveillance across the pork production continuum in uMgungundlovu District, KwaZulu-Natal Province, South Africa. Isolates were obtained from diverse sampling points, including pig feces, environmental slurry, water, cecal contents, carcass rinsates, transport truck surfaces, and retail meat cuts. The sampling design targeted critical control points along the farm-to-fork continuum: farm (n = 19), transport (n = 4), and abattoir (n = 8). Isolate selection was based on the following criteria: confirmed resistance to three or more classes of antibiotics, in accordance with CLSI definitions for multidrug resistance; phenotypic diversity based on distinct antibiogram profiles; representativeness across all three sampling interfaces; and the availability of high-quality genomic DNA suitable for whole-genome sequencing (WGS). Isolates with incomplete metadata, duplicate resistance profiles, or suboptimal DNA yield or quality were excluded from the final dataset.

#### 4.4.2. Genome Sequencing and Pre-Processing of Sequence Data

Thirty-one multidrug-resistant (MDR) isolates with diverse resistance profiles from various points along the farm-to-fork continuum were selected for whole-genome sequencing (WGS) and further characterization (Table S1). Genomic DNA (gDNA) was extracted and purified using the Gene Elute Bacterial genomic DNA kit (Sigma Aldrich, St. Louis, MI, USA) following the manufacturer’s instructions. Quantification and purification were undertaken using Nano Drop8000 (Thermo Scientific, Waltham, MA, USA) at 260/280 nm wavelength, with verification by agarose gel electrophoresis. The Nextera XT DNA preparation kit was used to prepare a pair-end library, and the Illumina MiSeq machine (Illumina, San Diego, CA, USA) was used for whole-genome sequencing. FASTQC (v0.12.0) was used to assess the quality of sequenced reads, followed by trimming using Sickle (v1.33; https://github.com/najoshi/sickle accessed on 15 November 2023). The high-quality reads were then assembled de novo using SKESA (v2.4.0; https://github.com/ncbi/SKESA accessed on 19 November 2023), a k-mer-based extension tool designed for accurate genome assembly [68].

Quality control thresholds were applied as follows: a minimum read depth of 30×, genome coverage ≥95%, and Phred quality score ≥ 30. Contigs shorter than 200 bp were excluded, and assemblies with total genome sizes below 4.4 Mb or above 6.4 Mb were discarded to avoid incomplete or potentially contaminated assemblies. The resultant contiguous sequences were submitted for gene annotation to GenBank using the NCBI Prokaryotic Genome Annotation Pipeline (PGAP).

#### 4.4.3. Molecular Typing of *E. coli* Isolates

Multilocus sequence typing (MLST) was performed in silico using the WGS data online platform MLST v2.0 (https://cge.food.dtu.dk/services/MLST/ accessed on 20 March 2024) based on the allelic profiles of the seven housekeeping genes (*adk*, *fumC*, *gyrB*, *icd*, *mdh*, *purA,* and *recA*) *of E. coli*, using the Achman scheme [69].

### 4.5. Resistome, Mobilome, and Genetic Support Analysis

To determine the antibiotic-resistant genes in the selected isolates, the generated contigs from the WGS data were analyzed by three different platforms to confirm and overcome the shortfalls of each platform. These platforms were the NCBI AMR finder (https://www.ncbi.nlm.nih.gov/pathogens/antimicrobial-resistance/AMRFinder/ accessed on 15 March 2024) as a high-quality, curated resistance gene database [70], the Comprehensive Antibiotic Resistance Database (CARD) (https://card.mcmaster.ca/analyze/rgi accessed on 22 May 2024) [71], and ResFinder (http://genepi.food.dtu.dk/resfinder accessed on 17 May 2024) [72]. PHAge Search Tool Enhanced Release (PHASTER; https://phaster.ca accessed on 25 May 2024) server was used to identify with default parameters, annotate, and visualize prophage sequences [73]. Insertion sequences and transposase in the genomes were predicted by blasting contigs on the ISFinder database (https://www-is.biotoul.fr/index.php accessed on 7 June 2024) [74]. Integrons were predicted by PGAP, and RAST subsystems were blasted on the INTEGRALL database to find the actual integrons [75].

### 4.6. Putative Virulome Analysis

Virulence determinants associated with *E. coli* were identified with the Virulence Finder 2.0 (https://cge.food.dtu.dk/services/VirulenceFinder/ accessed on 18 May 2024) [72].

### 4.7. Phylogenomic Analyses and Metadata Insights of the E. coli Isolates

CSI Phylogeny v1.2 (https://cge.food.dtu.dk/services/CSIPhylogeny-1.2/ accessed on 10 July 2024) was used to identify, filter, and validate SNPs from the de novo assembled contigs, and to construct a phylogenetic tree based on concatenated SNP profiles using default settings [76]. The genome of *Morganella morganii* subsp*. morganii KT* (Accession number: CP004345.1) served as the outgroup to root the tree, enabling the easy configuration of the phylogenetic distance between the strains on the branches. The phylogeny was visualized with annotations for isolate demographics, WGS in silico typing (ST), resistome, and MGE metadata to provide a comprehensive analysis of the generated phylogenomic tree.

Additionally, whole-genome sequences of *E. coli* isolates from South Africa (n = 31), along with curated sequences from the PATRIC database (https://www.patricbrc.org/ accessed on 28 June 2024) (n = 34) containing MLST, source, and geographical location data, were downloaded and analyzed alongside this study’s isolates. These sequences were used for whole-genome phylogeny analysis to assess the current epidemiological and evolutionary trends (Table S5). The Figtree v1.4.4 was used to edit and visualize the phylogenetic tree. Isolates of the same clade are highlighted in the same color, whilst those of the same countries have the same label (strain name) colors. The resistome of strains with a close phylogenetic relationship to this study’s isolates was searched using NCBI’s Pathogen Detection database (https://www.ncbi.nlm.nih.gov/pathogens/isolates#/search/ accessed on 30 June 2024).

### 4.8. Nucleotide Sequence

All nucleotide sequences of the *E. coli* strains from this study are available in the NCBI GenBank database under BioProject ID PRJNA596168.

## 5. Conclusions

The genomic analysis of *E. coli* isolates from an intensive pig production system in South Africa revealed substantial genetic diversity in antimicrobial resistance genes (ARGs), virulence factors, and mobile genetic elements (MGEs) across the farm-to-fork continuum. The widespread detection of plasmid-mediated β-lactamase genes (*bla_EC_*, *bla_TEM_*, *bla_AmpC_*), tetracycline resistance determinants (*tetA*, *tetB*, *tet34*), and integrons underscores the complexity and persistence of antimicrobial resistance in livestock environments. Importantly, the detection of virulence genes and co-localization of ARGs on transmissible elements raises concerns about the potential for zoonotic transfer and foodborne dissemination.

This study highlights the need for routine AMR surveillance integrated into food safety monitoring, particularly at critical control points like abattoirs and transport systems. Policymakers should prioritize the regulation of non-therapeutic antibiotic use in animal husbandry and support farmer education and biosecurity interventions. Future research should expand genomic surveillance across multiple farms and regions, with larger datasets and metadata to trace high-risk clones and better understand AMR transmission dynamics within a One Health framework.

## Figures and Tables

**Table 1:** Chromosomal mutations associated with resistance to fluoroquinolones and tigecycline.

Isolate ID	Source	Resistance Type	Genes	Amino Acid Substitutions	Associated Phenotype
A2-5-R3	Farm	Quinolone	*gyrA*, *gyrB*, *parC*, *parE*	S83L, D87N, E678D, S828A (***gyrA***); D185E (***gyrB***); E475D, A620V * (***parC***); I136V (***parE***)	Ciprofloxacin/Nalidixic acid
B4-6-R4	Farm	Quinolone	*gyrA*, *gyrB*, *parC*, *parE*	E678D, S828A (***gyrA***); D185E (***gyrB***); E475D (***parC***); I136V (***parE***)	Ciprofloxacin/Nalidixic acid
B4-9-R5	Farm	Quinolone	*gyrA*, *gyrB*, *parC*, *parE*	S83L, D87N, E678D, S828A (***gyrA***); D185E (***gyrB***); S80I, A58T *, E475D, A620V * (***parC***); I136V (***parE***)	Ciprofloxacin/Nalidixic acid
TB3-10	Transport	Quinolone	*gyrA*, *gyrB*, *parC*, *parE*	E678D, S828A (***gyrA***); D185E (***gyrB***); E475D (***parC***); I136V (***parE***)	Ciprofloxacin/Nalidixic acid
W1-7-R5	Farm	Quinolone	*gyrA*, *gyrB*, *parC*, *parE*	E678D, S828A (***gyrA***); D185E (***gyrB***); E475D (***parC***); I136V (***parE***)	Ciprofloxacin/Nalidixic acid
WA2-4-R1	Farm	Quinolone	*gyrA*, *gyrB*, *parC*, *parE*	S83L, D87N, E678D, S828A (***gyrA***); D185E (***gyrB***); A58T *, S80I, E475D, A620V * (***parC***); I136V (***parE***)	Ciprofloxacin/Nalidixic acid
B2-2-R1	Farm	Tigecycline	*acrA*, *acrR*, *marB*, *tolC*	T104A (***acrA***); A212S, N214T (***acrR***); L27P (***marB***); ***tolC*** present	Tigecycline

Note: * Novel mutations identified.

**Table 2:** Class 1 and 2 integrons, gene cassettes, and sequence types of the *E. coli* isolates.

Isolate ID	MLST ^a^	Integron Class	Integron	Cassette Arrays						
				GC1	GC2 ^b^	GC3	GC4	GC5	GC6	GC7
A1-10-R8	88	IntI1	In13	*-* ^c^	*dfrA5*	*-*	*-*	*-*	*-*	*-*
A2-4-R2	4373	IntI2	In2-32	*dfrA1*	*-*	*-*	*aadA1*	*-*	*-*	*sat2*
A5-1-1R4	10	IntI1	In456	*-*	*-*	*-*	*aadA1*	*aadA2*	*cmlA1*	*-*
W2-4-R8	10	IntI1	In649	*-*	*-*	*dfrA12*	*aadA1*	*aadA2*	*cmlA1*	*-*
W-5-R4	1109	IntI1	In456	*-*	*-*	*-*	*aadA1*	*aadA2*	*cmlA1*	*-*
TB3-10	3531	IntI1	In774	*-*	*-*	*-*	*-*	*-*	*cmlA1*	*-*
CAC1-7	206	IntI1	In127	*-*	*-*	*-*	*-*	*aadA2*	*-*	*-*
CAC1-8	1109	IntI1	In456	*-*	*-*	*-*	*aadA1*	*aadA2*	*cmlA1*	*-*

Note: ^a^ MLST denotes multilocus sequence type; ^b^ GC denotes gene cassettes; and ^c^ denotes the missing cassette arrays due to draft genomic sequences that were fragmented during the sequencing and assembling process into different contigs.

**Table 3:** Mobile genetic elements associated with antibiotic resistance genes in the isolates.

Isolate ID	Contig	Synteny of Resistance Genes and MGE	Plasmid/Chromosomal Sequence with Closest Nucleotide Homology (Accession Number)
A1-10-R8	6	*::::incFII::::IS26:dfrA5:IntI1:TnAs1*	*Escherichia coli strain* 18MD05VL07 005213EC plasmid pVPS18EC0676-1, complete sequence (CP063726.1)
A2-4-R2	28	*::::emrD::::mdtL::::IntI2:dfrA1:sat2:ant(3*″*)-Ia::::*	No significant similarity found
A2-5-R3	602	*tet(A):tetR(A)::aph(6)-Id:aph(3*″*)-Ib: sul2::::IS1::::*	*Escherichia coli* strain T28R chromosome, complete genome (CP049353.1)
	14	*::::floR::IS91*	*Escherichia coli* strain AH25 chromosome, complete genome (CP055256.1)
A2-10-R7	1	*::::TEM-1:Tn3*	*Escherichia coli* strain AMSCJX02 plasmid pAMSC5, complete sequence (CP031110.1)
A5-1-R4	60	*IS1::::aph(6)-Id:aph(3*″*)-Ib:ISVsa5:::: IS26:::sul3:IS256::ant(3*″*)-Ia:cmlA1::: IntI1::TnAs3::tetR(B):tet(B):tetC::*	*Salmonella enterica subsp. enterica serovar Indiana* strain SI67 plasmid pSI67-1, complete sequence (CP050784.1)
B1-1-R8	69	*:TEM-1:Tn3::::IS1*	*Escherichia coli* isolate MSB1_3C-sc-2280310 genome assembly, plasmid: 2 (LR890263.1)
B2-2-R1	171	*::::Tn3:::tet(A):tetR(A)::Tn3*	*Escherichia coli* O83:H1 str. NRG 857C plasmid pO83_CORR genomic sequence (CP001856.1)
CAC1-7	98	*mef(B)::::sul3::IS256:qacL:aadA1: cmlA1:ant(3*″*)-Ia:::intI1::TnAs1*	*Klebsiella pneumoniae* strain k9 plasmid pk9, complete sequence (CP049891.1)
CAC1-8-	79	*mef(B):::sul3:IS256::ant(3*″*)-Ia:cmlA1:ant(3*″*)-Ia:::IntI1:::TnAs1*	*Klebsiella pneumoniae* strain k9 plasmid pk9, complete sequence (CP049891.1)
W2-4-R8	127	*:::sul3:IS256::ant(3*″*)-Ia:cmlA1:ant(3*″*)-Ia::dfrA12:IntI1:TnAs3*	*Escherichia coli* strain 1919D3 plasmid p1919D3-1, complete sequence (CP046004.1)
W5R4	53	*IS26:mef(B):::sul3:IS256:qacL:aadA1: cmlA1:ant(3*″*)-Ia:::IntI1:TnAs1*	No significant similarity found
WA2-4-R1	145	*:aph(6)-Id:aph(3*″*)-Ib:sul2:::IS1::::*	*Escherichia coli* strain 13P484A chromosome, complete genome (CP019280.1)
	23	*::ISVsa3::floR::IS91*	*Escherichia coli* strain AH25 chromosome, complete genome (CP055256.1)
WB1-1-R8	59	*tetR(A):tet(A)::::TnAs1::::*	*Escherichia coli* TCJ482-1 plasmid p482-1 contig COV43U1_c1 genomic sequence (MG692709.1)
	57	*::::incFII::Tn3::TEM-1*	*Escherichia coli* strain ECOR 48 genome assembly, plasmid: RCS84_p (LT985305.1)
TB3-10	162	*:::sul3::IS256:::IntI1:::IS6*	*Escherichia coli* strain CP131_Sichuan plasmid pCP131-IncHI1, complete sequence (CP053721.1)
	376	*::tet(A):tetR(A)::TnAs1:TEM-1::*	*Klebsiella pneumoniae* strain 20130907-4 plasmid p309074-1FIIK complete sequence (MN842293.1)

**Table 4:** Mobile genetic elements associated with virulence genes.

Isolate ID	Contig	Virulence and MGE	Plasmid/Chromosomal Sequence with Closest Nucleotide Homology (Accession Number)
A1-10-R8	30	*:**ireA**:IS256:IS3::*	*Escherichia coli* strain Ecol_AZ159, complete genome (CP019008.1)
A2-4-R2	28	*::::**eilA**:::IntI2*	No significant similarity found
B5-3-R8	44	*IS3 family transposase::**tsh**::IS3 family transposase:IS30-like element IS30 family transposase:IS3 family transposase*	*Escherichia coli* strain CVM N55972 plasmid pN55972-1, complete sequence (CP043759.1)
CR2-3	36	*ISAs1 family transposase:::**gadC***	*Escherichia coli* strain CVM N18EC0432 chromosome, complete genome (CP048290.1)
CS4-4	19	*::**gadC**::::IS200/IS605 family transposase*	No significant similarity found
H-1-1	29	*::**gadx**::**gadE**::::IS5-like element ISKpn26 family transposase::*	*Escherichia coli* strain HS30-1 chromosome, complete genome (CP029492.1)
H2(12)	109	*::::IS110 family transposase::ISL3 family transposase:IS30-like element IS30 family transposase::**tsh***	*Escherichia coli* strain 14EC001 chromosome, complete genome (CP024127.1)
T3-9-		*:**gad**:::ISAs1::*	No significant similarity found
TA3-1	66	*:**stb**:IS3 family transposase*	*Escherichia coli* strain RHB38-C13 plasmid pRHB38-C13_2, complete sequence (CP055625.1)
	69	*::IS1 family transposase:ISAs1 family transposase::::**gad**:*	*Escherichia coli* strain EC11 chromosome, complete genome (CP027255.1)
TB3-2		*:**gad**:::ISAs1-like element ISEc1 family transposase*	*Escherichia coli* strain LD27-1 chromosome, complete genome(CP047594.1)
TB3-10	660	*IS3 family transposase:c**apU**:virK:IS30 family transposase*	*Escherichia coli* strain G4/9 chromosome, complete genome (CP060073.1)
W5-R4		***terC*** *::IS21 family transposase*	*Escherichia coli* PCN061, complete genome (CP006636.1)
WB1-1-R8	12	*::**gad**:::IS605 family transposase*	*Escherichia coli* strain 2 HS-C chromosome, complete genome (CP038180.1)

## Data Availability

The data presented in this study are openly available in National Center for Biotechnology Information [NCBI] GenBank database in the Bio-project number PRJNA596168 [https://www.ncbi.nlm.nih.gov/bioproject/?term=PRJNA596168 accessed on 24 April 2025].

## Supplementary Materials

The following supporting information can be downloaded at: https://www.mdpi.com/article/10.3390/antibiotics14050446/s1, Table S1: Population, specimen source, sample type, phenotypes and genotypic characteristics of the *E. coli* isolates; Table S2: Genomic characteristics of *E. coli* isolated along the farm-to-fork continuum; Table S3: Distribution of intact prophages among the *E. coli* isolates; Table S4: Distribution of virulence genes among *E. coli* isolates; Table S5: Metadata of *E. coli* sequences from South Africa (n = 34), downloaded and analyzed alongside this study’s isolates for whole-genome phylogeny analysis.
