# Supplementary material for: Genomic Insights of Antibiotic-Resistant Escherichia coli Isolated from Intensive Pig Farming in South Africa Using ‘Farm-to-Fork’ Approach
Source: Antibiotics (Basel). 2025 Apr 28;14(5):446. doi: 10.3390/antibiotics14050446 (PMC12108210; doi:10.3390/antibiotics14050446)
Supplement: Supplementary file 1 [file antibiotics-14-00446-s001.zip › antibiotics-3552322-supplementary.pdf]

Supplementary Materials

Genomic Insights of Antibiotic-Resistant *Escherichia coli* Isolated from Intensive Pig Farming in South Africa Using 'Farm-to-Fork' Approach

Shima E. Abdalla [1], Linda A. Bester [2], Akebe L. K. Abia [1,3], Mushal Allam [4,5], Arshad Ismail [5,6], Sabiha Y. Essack [1] and Daniel G. Amoako [1,7*]

[1] Antimicrobial Research Unit, College of Health Sciences, University of KwaZulu-Natal, Durban 4000, South Africa; shimaeltayeb23@gmail.com (S.E.A.); abiaakebel@ukzn.ac.za (A.L.K.A.);
essacks@ukzn.ac.za (S.Y.E.)

[2] Biomedical Resource Unit, College of Health Sciences, University of KwaZulu-Natal, Durban 4000, South Africa; besterl@ukzn.ac.za

[3] Environmental Research Foundation, Westville 3630, South Africa

[4] Department of Genetics and Genomics, College of Medicine and Health Sciences, United Arab Emirates University, Al Ain P.O. Box 15551, United Arab Emirates; mushal.allam@uaeu.ac.ae

[5] Sequencing Core Facility, National Institute for Communicable Diseases, Division of the National Health Laboratory Service, Johannesburg 2193, South Africa; arshadi@nicd.ac.za

[6] Department of Biochemistry and Microbiology, Faculty of Science, Engineering and Agriculture,
University of Venda, Thohoyandou 0950, South Africa

[7] Department of Pathobiology, University of Guelph, Guelph, ON N1G 2W1, Canada

* Correspondence: amoakod@ukzn.ac.za

Table S1: Population, specimen source, sample type, phenotypes and genotypic characteristics of the *E. coli* isolates

Isolate ID	Date	Source	Origin	ST	Antibiogram	Resistance genes	Plasmid replicons
A1-10-R8	27-Dec-18	Feacal	Farm	88	AMP-AMC-CHL-SXT-TE	bla_{EC-13}, bla_{AmpH}, bla_{AmpC1}, bla_{AmpC2}, *dfrA5*, *mdf(A)*	Col(MG828), IncFIB(AP001918), IncFII
A2-10-R4	01-Nov-18	Feacal	Farm	10	AMP-AMC-CHL-TE	bla_{EC}, bla_{AmpH}, bla_{AmpC1}, bla_{AmpC2}, *mdf(A)*, *tet(34)*	Col156, ColRNAI
A2-10-R7	13-Dec-18	Feacal	Farm	1286	AMP-AMC-FOX-CRO-CAZ-CTX-FEP-LEX-CHL-TE	bla_{EC-15}, bla_{TEM-1B}, $bla_{TEM-105}$, bla_{AmpH}, bla_{AmpC1}, bla_{AmpC2}, *mdf(A)*, *tet(34)*	Col(MG828),ColRNAI, IncFIB(AP001918),IncFIC(FII), IncFII_1_pSFO,IncX1, IncX3, IncY
A2-4-R2	04-Oct-18	Feacal	Farm	4373	AMP-AMC-AK-GEN-SXT-TE	bla_{EC-19}, bla_{AmpH}, bla_{AmpC1}, bla_{AmpC2}, *dfrA1*, *mdf(A)*, *ant(3'')-Ia*, *tet(34)*, *aadA*, *Sat2A*	Col8282,IncFIB(AP001918), IncFIC(FII),IncFII_1_pSFO,IncX1, IncX3, p0111
A2-5-R3	18-Oct-18	Feacal	Farm	542	AMP-AMC-CIP-NAL-CHL-GEN-SXT-TE	bla_{EC}, bla_{AmpH}, bla_{AmpC1}, bla_{AmpC2}, *sul3*, *sul2*, *aph(6)-Id*, *tet(A)*, *tet(R)*, *floR*, *aph(3'')-Ib*, *aph(6)-Id*, *strA*, *strB*,	Col(MG828), IncFII(pCRY)_1_pCRY, IncR, IncX1, IncX3, IncX4, IncY
A3-10-R4	01-Nov-18	Feacal	Farm	10	AMP-AMC-CHL-TE	bla_{EC}, bla_{AmpH}, bla_{AmpC1}, bla_{AmpC2}, *mdf(A)*, *tet(34)*	Col156, ColRNAI
A5-1-R4	01-Nov-18	Feacal	Farm	10	AMP-AMC-LEX-CHL-AK-GEN-AZM-SXT-TE	bla_{EC}, bla_{AmpC1}, bla_{AmpC2}, *sul3*, *mdf(A)*, *ant(3'')-Ia*, *tet(34)*, *tet(A)*, *tet(B)*,*cmlA1*, *strA*, *strB*, *aadA1*, *aadA2*, *aph(6)-Id*, *aph(3'')-Ib*, *qacL*, *emrE*	IncFIB(AP001918), IncFIC(FII), IncFII_1_pSFO
A5-5-R3	18-Oct-18	Feacal	Farm	10	AMP-AMC-LEX-CHL-TE	bla_{EC-15}, bla_{AmpH}, bla_{AmpC1}, *blaAmpC2*, *mdf(A)*, *tet(34)*, *catB4*	Col(MG828), ColRNAI, IncFIB(AP001918), IncFII_1_pSFO, IncX1
B1-1-R8	27-Dec-18	Feacal	Farm	58	AMP-AMC-LEX-FOX-FEP-CRO-CAZ-CTX-CHL-TE	bla_{EC-18}, bla_{TEM-1B}, $bla_{TEM-105}$, bla_{TEM-1}, bla_{AmpH}, bla_{AmpC1}, bla_{AmpC2}, *mdf(A)*, *tet(34)*	Col(MG828), IncI1_1_Alpha, IncN, IncX1, IncX3
B2-2-R1	19-Sep-18	Feacal	Farm	117	AMP-AMC-FOX-CRO-LEX-AZM-TE-TGC	bla_{EC}, bla_{AmpH}, bla_{AmpC}, bla_{AmpC2}, *mdf(A)*, *mphB*, *tet(A)*, *TetR*	Col(MG828), ColRNAI, IncFIA, IncFIB(AP001918), IncFIB(pKPHS1)_1_pKPHS1, IncFII_1_pSFO, IncI1_1_Alpha
B3-1-8	27-Dec-18	Feacal	Farm	165	AMP-AMC-LEX-CHL-TE	bla_{EC}, bla_{AmpH}, bla_{AmpC1}, bla_{AmpC2}, *mdf(A)*, *tet(34)*	Col(MG828), ColRNAI, IncFIA, IncFIB(AP001918), IncFIC(FII), IncFII(29)_1_pUTI89,IncFII_1_pSFO, IncI1_1_Alpha

B4-6-R4	01-Nov-18	Feacal	Farm	10	AMP-AMC-LEX-FOX-CRO-CIP-NAL-CHL-TE	*bla*$_{EC}$, *bla*$_{AmpH}$, *bla*$_{AmpC1}$, *bla*$_{AmpC2}$, *mdf(A)*, *tet(34)*	ColRNAI
B4-9-R5	15-Nov-18	Feacal	Farm	542	AMP-AMC-LEX-FOX-CRO-AZM-TE	*bla*$_{EC}$, *bla*$_{AmpH}$, *bla*$_{AmpC1}$, *bla*$_{AmpC2}$, *mdf(A)*, *tet(34)*, *emrE*	Col(MG828), ColRNAI, IncX1, IncX4, IncY
B5-3-R8	27-Dec-18	Feacal	Farm	206	AMP-AMC-CHL-TE	*bla*$_{EC}$, *bla*$_{AmpH}$, *bla*$_{AmpC1}$, *bla*$_{AmpC2}$, *mdf(A)*, *tet(34)*	Col(MG828),IncFIB(AP001918), IncFIC(FII), IncFII_1_pSFO
W1-7-R5	15-Nov-18	water	Farm	641	AMP-AMC-AZM-LEX-FOX-CHL-TE	*bla*$_{EC-13}$, *bla*$_{AmpH}$, *bla*$_{AmpC1}$, *bla*$_{AmpC2}$, *tet(34)*, *emrE*	Col8282, IncFIB(AP001918), IncFIC(FII), IncFII_1_pSFO, IncR, IncX1
W2-4-R8	27-Dec-18	water	Farm	10	AMP-AMC-CHL-AK-GEN-AZM-SXT-TE-	*bla*$_{EC-15}$, *bla*$_{AmpH}$, *bla*$_{AmpC1}$, *bla*$_{AmpC2}$, *sul3* ,*dfrA12*, *mdf(A)*, *mefB*, *ant(3'')-Ia*, *tet(34)*, *cmlA1*, *aac3-Ib*, *aadA2*, *qacl*	ColRNAI, IncFIB(AP001918), IncFII_1_pSFO, IncR, IncX1
W-5-R4	01-Nov-18	water	Farm	1109	AMP-AMC-LEX-FOX-CRO-AZM-CHL-GEN-SXT-TE	*bla*$_{EC}$, *bla*$_{AmpH}$, *bla*$_{AmpC1}$, *bla*$_{AmpC2}$, *sul3*, *mdf(A)*, *mefB*, *ant(3'')-Ia* *tet(34)*, *cmlA1*, *aadA1*, *aadA2*, *qacl*, *emrE*	Col(MG828), Col156, ColRNAI, IncY
WA2-4-R1	19-Sep-18	water	Farm	542	AMP-AMC-CIP-NAL-CHL-GEN-SXT-TE	*bla*$_{EC}$, *bla*$_{AMPH}$, *bla*$_{AmpC1}$, *bla*$_{AmpC2}$, *sul2*, *mdf(A)*, *tet(34)*, *floR*, *strA*, *strB*, *aph(6)-Id*, *aph(3'')-Ib*,	ColRNAI, IncX1, IncX4, IncY
WB3-1-R7	13-Dec-18	water	Farm	641	AMP-CHL-TE	*bla*$_{EC-13}$, *bla*$_{AmpH}$, *bla*$_{AmpC2}$, *mdf(A)*, *tet(34)*	ColRNAI, IncFIB(AP001918), IncFII
WB1-1-R8	27-Dec-18	water	Farm	6354	AMP-AMC-FOX-CRO-CAZ-CTX-FEP-LEX-MEM-IPM-CHL-TE	*bla*$_{EC-15}$, *bla*$_{TEM-1B}$, *bla*$_{TEM-1}$, *bla*$_{TEM-105}$, *bla*$_{AmpH}$, *bla*$_{AmpC1}$, *bla*$_{AmpC2}$, *mdf(A)*,	ColRNAI, IncFIB(pB171)_1_pB171, IncFII, IncFII_1_pSFO , IncI1_1_Alpha
TA2-6	16-Jan-19	Truck after	Truck	877	AMP-AMC-LEX-FOX-CRO-AK-GEN-TE	*bla*$_{EC-13}$, *bla*$_{AmpH}$, *bla*$_{AmpC1}$, *bla*$_{AmpC2}$, *mdf(A)*, *aadA1*	ColRNAI,IncFIB(AP001918), IncFIC(FII),IncFII_1_pSFO, IncY
TA3-1	16-Jan-19	Truck after	Truck	336	AMP-AMC-AZM-TE	*bla*$_{EC-18}$, *bla*$_{AmpH}$, *bla*$_{AmpC1}$, *bla*$_{AmpC2}$, *mdf(A)*, *tet(34)*, *emrE*	ColRNAI, IncFIB(AP001918), IncFII, IncX1 , IncX3
TB3-10	16-Jan-19	Truck before	Truck	3531	AMP-AMC-FOX-CRO-CAZ-CTX-FEP-LEX--CIP-NAL-CHL-SXT-TE	*bla*$_{EC}$, *bla*$_{EC-15}$, *bla*$_{AmpC1}$, *bla*$_{AmpC2}$, *bla*$_{AmpH}$, *bla*$_{TEM-1}$, *sul3*, *tet-34*, *tetR*, *tetA*, *catB4*, *cmlA1*, *qacl*	IncFIB(AP001918), IncFIC(FII), IncFII_1_pSFO
TB3-2	16-Jan-19	Truck before	Truck	10	AMP-AMC-CHL-TE	*bla*$_{EC}$, *bla*$_{TEM-1}$, *bla*$_{TEM-1B}$, *bla*$_{TEM-105}$, *bla*$_{AmpC1}$, *bla*$_{AmpC2}$, *sul3*, *mdfA*, *tet(R)*, *tet(34)*, *tet(A)*, *cmlA1*, *catB4*	No plasmid found

CAC1-7	17-Jan-19	caecal	Abattoir	206	AMP-AMC-CHL-AK-GEN-TE-SXT	bla_{AmpC1}, bla_{AmpH}, *sul3*, *tet(B)*, *tet(A)*, *tet(R)*, *aadA2*	Col(BS512), ColpVC, IncFIC(FII), IncX1
CAC1-8	17-Jan-19	caecal	Abattoir	1109	AMP-AMC-CHL-GEN-AZM-SXT-TE	bla_{EC}, bla_{AmpH}, bla_{AmpC1}, bla_{AmpC2}, *sul3*, *mdf(A)* ,*mef(B)*, *ant(3'')-Ia*, *tet(34)*, *cmlA1*, *aadA1*, *aadA2*, *qacl*	Col(MG828), IncY
CR1-2	17-Jan-19	carcass rinsate	Abattoir	898	AMP-AMC-CHL-GEN-TE	bla_{EC-18}, bla_{AmpH}, bla_{AmpC1}, bla_{AmpC2}, *mdf(A)*, *tet(34)*, *aph(3'')-Ib*, *strB*, *strA*, *aph(6)-Id*	ColRNAI, IncFIB(AP001918), IncFIC(FII), IncFII_1_pSFO ,IncX1, IncY
CR2-3	17-Jan-19	carcass rinsate	Abattoir	453	AMP-AMC-CHL-TE	bla_{EC-13}, bla_{AmpH}, bla_{AmpC1}, bla_{AmpC2}, *mdf(A)*, *tet(34)*	Col8282, ColRNAI, IncR
CS4-4	17-Jan-19	carcass swab	Abattoir	101	AMP-LEX-CHL-TE	bla_{EC-18}, bla_{AMPH}, bla_{AmpC1}, bla_{AmpC2}, *mdf(A)*, *tet(34)*	Col(MG828), IncFIB(AP001918),IncFIC(FII),IncFII_1_pSFO,IncI1_1_Alph h
H2-2-12	18-Jan-19	meat cut	Abattoir	101	AMP-AMC-CHL-TE	bla_{EC-18}, bla_{AmpH} , bla_{AmpC1}, bla_{AmpC2}, *mdf(A)*, *tet(34)*	Col(MG828)_1,ColRNAI, IncFIB(AP001918) , IncFIC(FII)_1, IncFII_1_pSFO, IncI1_1_Alpha
T3-9	18-Jan-19	meat cut	Abattoir	5876	AMP-AMC-LEX-CHL-TE	bla_{EC}, bla_{AmpH}, bla_{AmpC1}, bla_{AmpC2}, *mdf(A)*, *tet(34)*	Col8282, ColRNAI, IncB/O/K/Z_4, IncFIC(FII), IncFII_1_pSFO

Key: β-lactams made up of penicillins [ampicillin (AMP)], penicillin-inhibitor combinations [amoxicillin-clavulanate (AMC), piperacillin-tazobactam (TZP)], cephalosporins [cephalexin (LEX), ceftriaxone (CRO), cefotaxime (CTX), cefepime (FEP), ceftazidime (CAZ)], cephamycins, [cefoxitin (FOX)], carbapenems [meropenem (MEM), and imipenem (IMP)], macrolides [azithromycin (AZM)], aminoglycosides [gentamicin (GEN), amikacin (AMK)], tetracyclines [tetracycline (TET), tigecycline (TGC)], amphenicols [chloramphenicol (CHL)], sulphonamides [trimethoprim-sulfamethoxazole (SXT)], and quinolones [ciprofloxacin (CIP), nalidixic acid (NAL)].

TABLE. S2. Genomic characteristics of *E. coli* isolated along the farm to fork continuum

Isolate ID	Accession no.	Size (Mb)	GC%	Contigs	No. of RNAs	No. of coding sequences	N_{50}	L_{50}	Coverage (X)	No. of CRISPR arrays
**FARM**										
A1-10-R8	JAAAGH000000000.1	5,012,393	50.7	136	84	5013	137,228	13	99	1
A2-10-R4	JAAAGI000000000.1	4,635,407	50.8	83	58	4580	124,914	13	99	2
A2-10-R7	JAAAGJ000000000.1	4,991,884	50.6	78	87	4995	132,189	13	99	1
A2-4-R2	JAAAGK000000000.1			75	91		144,571	10	99	1
A2-5-R3	JAAAGL000000000.1	5,203,988	50.3	783	76	5910	12,100	135	99	4
A3-10-R4	JAAAGM000000000.1	4,637,120	50.8	79	88	4587	130,908	12	99	2
A5-1-R4	JAAAGN000000000.1	4,774,234	50.6	71	92	4759	147,489	11	99	3
A5-5-R3	JAAAGO000000000.1	4,765,135	50.7	113	93	4753	116,935	16	99	2
B1-1-R8	JAAAGP000000000.1	4,930,774	50.7	75	88	4922	133,288	12	99	2
B2-2-R1	JAAAGQ000000000.1	6,135,044	50.4	570	104	6621	84,635	22	99	3
B3-1-8	JAAAGR000000000.1	5,308,519	50.6	147	101	5549	102,741	16	99	1
B4-6-R4	JAAAGT000000000.1	4,637,156	50.8	75	93	4589	144,535	12	99	2
B4-9-R5	JAAAGU000000000.1	4,851,259	50.6	107	84	4889	103,396	17	99	3
B5-3-R8	JAAAGV000000000.1	4,719,387	50.6	72	82	4636	107,695	12	99	3
W1-7-R5	JAAAHK000000000.1	5,038,523	50.2	797	61	5543	13,922	104	99	2
W2-4R8	JAAAHL000000000.1	4,912,338	50.7	103	85	4903	97,385	16	99	2
W5-R4	JAAAHM000000000.1	4,834,216	50.7	91	83	4776	135,203	14	99	2
WA2-4R1	JAAAHN000000000.1	4,893,553	50.5	115	84	4959	102,725	17	99	3
WB3-1R7	JAAAHP000000000.1	5,090,538	50.8	130	96	5270	91,550	19	99	2
WB1-1-R8	JAAAHQ000000000.1	4,796,748	50.7	127	86	4611	79,857	12	99	3
**TRUCK**										
TA2-6	JAAAHE000000000.1	6,100,096	50.7	1467	86	7178	8358	200	99	4
TA3-1	JAAAHG000000000.1	4,937,266	50.5	79	90	4955	161,561	10	99	2

TB3-10	JAAAHI000000000.1	5,568,062	50.5	728	79	6026	21,795	75	99	2
TB3-2	JAAAHJ000000000.1	4,632,194	50.8	74	78	4572	124,924	13	99	2
**ABATTOIR**										
CAC1-7	JAALJG000000000.1	4,769,860	50.6	663	76	5176	13,319	106	99	3
CAC1-8	JAAAGX000000000.1	5,568,062	50.5	728	79	6026	21,795	75	99	2
CR1-2	JAAAGY000000000.1	5,100,723	50.5	1198	75	5884	9,113	142	99	2
CR2-3	JAAAGZ000000000.1	5,010,763	50.5	106	86	5038	101,917	17	99	2
CS4-4	JAAAHA000000000.1	4,836,500	50.6	44	80	4786	305,461	5	99	2
H2-2	JAAAHC000000000.1	4,954,211	50.6	269	79	5076	62,855	28	99	1
T3-9	JAAAHD000000000.1	5,221,771	50.5	50	92	5132	521,884	4	99	2

Table S3: Distribution of intact prophages among the *E. coli* isolates

ISOLATE ID	REGION [a]	REGION_LENGTH [b]	#CDS [c]	INTACT PHAGE (hit genes count) [d]
A1-10- R8	1	35.5Kb	44	Entero_SfI
	2	33.8Kb	48	Entero_mEp460
	3	45.1Kb	22	Entero_mEp460
	4	20.2Kb	24	Pseudo_phiPSA1
	5	23.5Kb	24	Entero_lambda
A2-4-R2	1	56.7Kb	61	Pectob_ZF40
	2	110.1Kb	130	Entero_P1
A2-5-R3	1	26.2Kb	38	Entero_P88
	2	28.4Kb	35	Gordon_BritBrat
	3	33.1Kb	27	Escher_pro483
	4	31.5Kb	40	Entero_lambda
	5	40.1Kb	44	Escher_phiV10
	6	44.7Kb	62	Entero_lambda
	7	33.4Kb	32	Entero_WPhi
	8	34.9Kb	40	Salmon_Fels
	9	93.8Kb	107	Entero_SfV
A2-10-R4	1	34.9Kb	36	Salmon_Fels
	2	46.9Kb	47	Entero_P88
	3	38.8Kb	15	Entero_P4
	4	45.5Kb	36	Entero_P2
	5	45.2Kb	52	Shigel_SfII
A2-10-R7	1	56.7Kb	61	Entero_P1
	2	22.3Kb	23	Entero_BP_4795
	3	64.1Kb	72	Salmon_SEN34
	4	19.7Kb	14	Entero_P4
	5	37.2Kb	32	Shigel_SfIV
	6	32.4Kb	25	Shigel_SfII
	7	29.2Kb	37	Salmon_Fels
	8	39.3Kb	34	Escher_pro147
A3-10-R4	1	32.3Kb	12	Entero_BP_4795
	2	41.5Kb	37	Salmon_Fels_2
	3	38.8Kb	14	Entero_P4
	4	35.5Kb	35	Entero_P88
	5	45.5Kb	36	Entero_P2
	6	45.2Kb	51	Shigel_SfII
A5-1-R4	1	32.1Kb	46	Salmon_Fels_2
	2	38.3Kb	48	Salmon_SEN34
A5-5-R3	1	27.9Kb	35	Entero_lambda
	2	40.1Kb	33	Salmon_Fels_2
	3	44.1Kb	40	Entero_P88
	4	14.6Kb	21	Salmon_Fels_2

B1-1-R8	1	49.9Kb	56	Entero_lambda_
	2	36.3Kb	53	Entero_P88
B2-2-R1	1	98.6Kb	111	Salmon_SSU5_NC
	2	34.4Kb	23	Shigel_SfII
	3	20.3Kb	25	Entero_BP_4795
	4	38.5Kb	49	Entero_P88
	5	26.4Kb	31	Entero_mEp460
B3-1-R8	1	27.2Kb	28	Pseudo_phiPSA1
	2	73.2Kb	75	Entero_mEp460
	3	15.3Kb	15	Stx2_c_1717
	4	49.2Kb	58	Entero_lambda
	5	22.9Kb	33	Salmon_SJ46
	6	28.7Kb	39	Entero_BP_4795
	7	20Kb	34	Entero_VT2phi_272
	8	35Kb	37	Salmon_Fels_2
B4-6-R4	1	50Kb	44	Salmon_Fels
	2	46.9Kb	47	Entero_P88
	3	38.8Kb	15	Entero_P4
	4	44.6Kb	36	Entero_P2
	5	45.2Kb	51	Shigel_SfII
B4-9-R5	1	41.9Kb	55	Escher_phiV10
	2	20Kb	32	Entero_WPhi
	3	46.9Kb	60	Entero_lambda
	4	42.4Kb	45	Shigel_SfII
	5	57.2Kb	60	Entero_SfV
B5-3-R8	1	61.4Kb	62	Entero_lambda
	2	22.8Kb	17	Stx2_II
	3	54.3Kb	44	Entero_mEp460
	4	29.1Kb	38	Entero_fiAA91_ss
WA2-4-R1	1	41.9Kb	56	Escher_phiV10
	2	39.8Kb	58	Shigel_Sf6
	3	51.8Kb	58	Entero_P1
	4	44.2Kb	33	Entero_WPhi
	5	61.2Kb	61	Entero_lambda
	6	47.9Kb	50	Shigel_SfII
	7	52.4Kb	60	Entero_SfV
W-5-R4	1	96.1Kb	95	Entero_P1
	2	22.3Kb	24	Shigel_SfII
	3	33.5Kb	33	Escher_pro147
	4	37.6Kb	42	Entero_lambda
W1-7-R5	1	23.9Kb	21	Salmon_RE_2010
	2	33.8Kb	27	Shigel_SfII
WB-1-R7	1	10.9Kb	11	Entero_P4
	2	32.2Kb	44	Salmon_Fels_2

	3	18.1Kb	22	Escher_D108
	4	44.1Kb	50	Entero_P88
	5	24.9Kb	30	Entero_P88
	6	25.9Kb	32	Salmon_118970_sal4
	7	13.6Kb	17	Pseudo_PPpW_3
	8	65.2Kb	97	Vibrio_12B12
	9	24.8Kb	35	Entero_c_1_
	10	27.4Kb	29	Vibrio_X29
	11	27.7Kb	14	Entero_lambda
	12	51.7Kb	64	Entero_lambda
W2-4-R8	1	45.4Kb	58	Escher_TL_2011b
	2	17.2Kb	19	Entero_lambda
	3	41.7Kb	37	Salmon_Fels_2
	4	22Kb	28	Salmon_118970_sal4
	5	16.1Kb	19	Salmon_Fels_2
	6	27.7Kb	32	Entero_P2
	7	46.3Kb	67	Salmon_SJ46
WB1-1-R8	1	42.7Kb	41	Entero_P2
	2	23.2Kb	25	Entero_lambda
	3	14.9Kb	17	Entero_HK630
TA2-6	1	21.8Kb	30	Salmon_RE_2010
	2	9.7Kb	16	Entero_P88
	3	12.1Kb	17	Entero_P88
	4	34.8Kb	32	Entero_mEp460
TA3-1	1	83.9Kb	111	Entero_UAB_Phi20
	2	6.1Kb	10	Entero_HK630
	3	9.8Kb	16	Entero_P1
	4	64.2Kb	72	Entero_lambda
	5	91.8Kb	112	Entero_P1
TB3-2	1	46.9Kb	47	Entero_P88
	2	38.8Kb	16	Entero_P4
	3	75.5Kb	59	Salmon_Fels_2
	4	44.6Kb	36	Entero_P2
	5	45.2Kb	52	Shigel_SfII
TB3-10	1	15.4Kb	19	Entero_HK630
	2	30.2Kb	40	Salmon_Fels_2
CAC1-7	1	45.4Kb	59	Entero_lambda
	2	38.6Kb	35	Entero_fiAA91_ss
	3	21.2Kb	23	Shigel_SfII
CAC1-8	1	96.1Kb	95	Entero_P1
	2	55.1Kb	53	Entero_SfV
	3	21.6Kb	25	Shigel_SfII
	4	33.5Kb	33	Escher_pro147
	5	13.7Kb	16	Escher_pro483
	6	37.7Kb	47	Entero_lambda

CR1-2	1	29.7Kb	35	Entero_P88
	2	36.6Kb	56	Entero_SfI_
	3	37.6Kb	49	Entero_lambda
CR2-3	1	27.2Kb	29	Entero_lambda
	2	20.2Kb	32	Salmon_SJ46
	3	29.1Kb	33	Entero_HK629
	4	23.8Kb	25	Entero_mEp460
	5	34.5Kb	50	Entero_HK629
	6	24.6Kb	35	Salmon_Fels_2
CS4-4	1	40.5Kb	38	Salmon_Fels_2
	2	35.7Kb	45	Entero_fiAA91_ss
	3	40.7Kb	58	Entero_SfV
H2-2-12	1	40.7Kb	58	Entero_SfV
	2	31.9Kb	41	Entero_fiAA91_ss
	3	22.2Kb	32	Salmon_Fels_2
T3-9	1	39.9Kb	57	Entero_SfMu
	2	57.8Kb	73	Entero_lambda
	3	26.6Kb	32	Entero_BP_4795
	4	35.2Kb	36	Entero_mEp460
	5	17.4Kb	21	Stx2_c_1717

[a] Intact prophage region; [b] Region length of intact prophage; [c] Number of CDS in the region of the phage; [d] Predicted intact prophage.

`

Table S4: Distribution of virulence genes among *E. coli* isolates

Isolate ID	Source	ST	Toxin	Adhesion	Iron uptake	Immunity	Microcin	Others
A1-10-R8	Farm	88		*lpfA*	*iroN, ireA*	*iss*	*mchF*	
A2-10-R4	Farm	10	*astA*			*iss*		
A2-10-R7	Farm	1286		*tsh*				
A2-4-R2	Farm	4373		*eilA, lpfA*		*iss, gad, air*		
A2-5-R3	Farm	542						
A3-10-R4	Farm	10	*astA*			*iss*		
A5-1-1R4	Farm	10						
A5-5-R3	Farm	10		*tsh,*	*iroN*			
B1-1-R8	Farm	58		*lpfA*		*gad*		
B2-2-R1	Farm	117	*vat, pic*	*iha, lpfA*	*iroN, ireA*	*iss*	*mchB, mchC, mchF*	*cma*
B3-1-8	Farm	165	*astA*					*aaiC, katP, cba*
B4-6-R4	Farm	10	*astA*			*iss*		
B4-9-R5	Farm	542						
B5-3-R8	Farm	206	*astA*	*tsh*				
W1-7-R5	Farm	641		*lpfA*		*iss*		
W2-4-R8	Farm	10			*iroN*			
W5R4	Farm	1109						
WA2-4-R1	Farm	542						
WB3-1-R7	Farm	641		*lpfA, stx2A, stx2B*		*gad*		
WB1-1-R8	Farm	6354				*gad*		
TA2-6	Truck	877		*lpfA*				
TA3-1	Truck	336	*astA*			*stb, iss, gad*		
TB3-10	Truck	3531	*capU*					
TB3-2	Truck	10	*astA*			*gad, iss*		
CAC1-8	Abattoir	1109						
CR1-2	Abattoir	898		*lpfA*		*gad*		
CR2-3	Abattoir	453		*lpfA*		*mchB, gad, iss*	*mchC, mchF*	*mcmA*

CS4-4	Abattoir	101		*lpfA,tsh*	*iroN*	*iss*	*mchF*	
H2-2-12	Abattoir	101		*lpfA, tsh*	*iroN*	*iss*	*mchF,*	
T3-9	Abattoir	5876	*astA*	*eilA*		*espP, gad, air, iss*		*katP*
CAC1-7	Abattoir	206	*astA*		*iroN*	*gad, iss*	*mchF*	

a. Empty rows indicate the absence of virulence factors.

Table S5: Metadata of *E. coli* sequences from South Africa (n=34), downloaded and analyzed alongside this study's isolates for whole-genome phylogeny analysis.

Genome ID	MLST	Isolation Country	Geographic Location
562.54054	ST1193	South Africa	WESTREN CAPE
562.54058	ST1193	South Africa	WESTRN CAPE
562.54056	ST1193	South Africa	WESTRN CAPE
562.54061	ST1193	South Africa	WESTRN CAPE
562.54059	ST636	South Africa	WESTREN CAPE
562.54060	ST636	South Africa	WESTREN CAPE
562.20528	ST95	South Africa	GAUTENG
562.20530	ST73	South Africa	GAUTENG
562.21492	ST998	South Africa	GAUTENG
562.20534	ST998	South Africa	GAUTENG
562.20525	ST131	South Africa	GAUTENG
562.20529	ST131	South Africa	GAUTENG
562.20531	ST131	South Africa	GAUTENG
562.20533	ST131	South Africa	GAUTENG
562.20535	ST131	South Africa	GAUTENG
562.21892	ST131	South Africa	GAUTENG
562.21893	ST131	South Africa	GAUTENG
562.21894	ST131	South Africa	GAUTENG
562.54063	ST131	South Africa	WESTREN CAPE
562.21493	ST131	South Africa	GAUTENG
562.21896	ST131	South Africa	GAUTENG
562.54055	ST131	South Africa	WESTREN CAPE
562.54057	ST131	South Africa	WESTREN CAPE
562.13800	ST6	South Africa	South Africa
562.21494	ST410	South Africa	GAUTENG
562.17753	ST21	South Africa	South Africa
562.54062	ST602	South Africa	WESTREN CAPE
562.20526	ST665	South Africa	GAUTENG
562.54064	ST1491	South Africa	WESTREN CAPE
562.20532	ST744	South Africa	GAUTENG
562.20527	ST617	South Africa	GAUTENG
562.21895	ST617	South Africa	GAUTENG
562.50308	ST641	South Africa	KWAZULU-NATAL
562.20536	ST648	South Africa	GAUTENG
